# Model and simulator of inlet air flow in grinding installation with electromagnetic mill

**DOI:** 10.1038/s41598-023-34664-0

**Published:** 2023-05-22

**Authors:** Oliwia Krauze

**Affiliations:** Department of Measurements and Control Systems, Faculty of Automatic Control, Electronics and Computer Science, Silesian University of Technology, 44-100 Gliwice, Poland

**Keywords:** Engineering, Applied mathematics, Scientific data, Software

## Abstract

Comminution of raw materials consumes great shares of energy and operating costs of production and processing plants. Savings may be achieved, e.g., by developing new grinding equipment, such as the electromagnetic mill with its dedicated grinding installation; and by applying efficient control algorithms to these elements. Good quality control relies on mathematical models, and testing of versatile control algorithms is much simplified if a plant simulation environment is available. Thus, in this research, measurements were collected at the grinding installation with electromagnetic mill. Then, a model was developed that characterised the flow of transport air in the inlet part of the installation. The model was also implemented in software to provide the pneumatic system simulator. Verification and validation tests were conducted. They confirmed the correct behaviour of the simulator and good compliance with the experimental data, for both steady-states and transients. The model is then suitable for design and parametrization of air flow control algorithms and for their testing in simulation.

## Introduction

Comminution of raw materials is a vital part in multiple industry branches, being a crucial stage in: food processing; manufacturing of paper, pharmaceuticals, cosmetics, pigments; mineral material treatment (for metallurgy, building construction, chemical and energetic sectors); waste recycling; and more. It is also a massive-scale process. For instance, global copper mine production reached 21.2 million tonnes of pure metal in 2021^1^. With copper ores being low grade—on average, there was 0.65% copper content in the material mined in 2015^2^—this means that a colossal quantity of over 3.2 billion tonnes of copper ore was mined, crushed and ground in just one year. Being such a common and large-scale process, comminution consumes close to 2% of global electrical energy^3^. Also, it often constitutes a very significant share in energy consumption and expenses on a mining or production site. For instance, at mines, the comminution and particle separation processes typically constitute about 30–50% of overall plant energy usage^4^ and about 35–55% of its operational costs^5^.

Reduction of expenses, energy consumption, and environmental impact of industrial processes is generally desired and continuously drives the innovation in grinding technologies^6^. This means: development of new grinding and particle classification equipment^7^; or applying more efficient control schemes to the existing solutions^8^; or extra treatment of the raw material—with chemical additives^9^, cold^10^, heat, microwaves, ultrasounds, high voltage, and others^7,11^. New mill types are being invented especially for fine and ultra-fine grinding, where conventional tumbling mills are ineffective or energetically inefficient^7^. A comparison of numerous mill designs, such as tumbling (ball, rod, autogenous), roller, stirred, vibratory, centrifugal, jet (fluid energy) mills may be found e.g. in^12–14^.

One of recent inventions in ultra-fine grinding is an electromagnetic mill^15–18^. It includes an inductor of strong rotating electromagnetic field, which moves small ferromagnetic rods (grinding elements) and causes very fast grinding or mixing of supplied raw materials. Feed particles are subjected to high impact of moving grinding elements, but also to heat, electric, magnetic and acoustic stresses, which further help to develop raw material fractures^15^. Maximum particle size of the feed material is about 1–2 mm, depending on the diameter of the mill’s working chamber. After grinding, the product particles are sized about tens of micrometers, depending on material type, particle size distribution of the feed, grinding time, and other operating conditions^19^.

To better leverage the device’s potential, a grinding system was designed, patented and built^19–21^. The installation includes underpressure transport of the processed material, particle classification and recycle, a dedicated measurement system and a layered control system. This setup incorporates the mill with vertically positioned working chamber. Such solution ensures flexibility in controlling the mill throughput and product’s particle size; however, at the same time it requires precise control of the transport air flow^19,22,23^. This research identifies models of air flow in the inlet part of the installation and provides a simulation environment for easier testing of various air flow control schemes. Moreover, the models developed here will serve as a basis for proper tuning of these control algorithms.

Some air flow models for this grinding installation were already presented in literature. Papers^22,24^ examined only the steady state flows, not the transient behaviour, as they aimed at supervisory layer control (i.e., second layer, counting from the bottom of the hierarchy). Paper^23^ identified both static and dynamic characteristics, but only for one air stream. The current paper presents both steady-state and dynamic parameters for all three streams, allowing to design, parametrize and test the algorithms in supervisory and also direct control layers. Furthermore, processing of experimental data is improved compared to these previous works. Namely, air flow is estimated more accurately from air speed; pressure models are also identified; more stages of outlier detection and removal are applied; and the calculated coefficients are interpolated to the whole operating range of air dampers. Moreover, model parameters are not only estimated, but also combined into one structure which was actually implemented in code, to form a complete simulation model of the inlet air flow and pressure. Correctness of such simulator is then verified.

## Methods

### Test rig—grinding installation with electromagnetic mill

**Figure 1:**
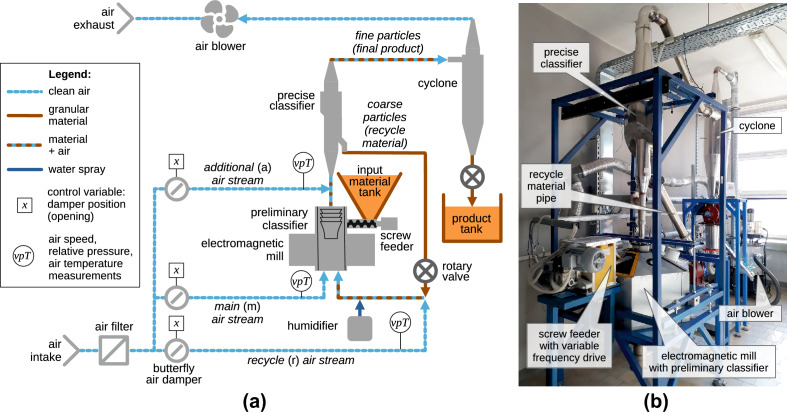
Dry grinding installation with electromagnetic mill: (**a**) diagram, (**b**) photo.

The grinding installation used in this research is shown in Fig. 1. Feed material is supplied with a screw feeder and enters the working chamber of the mill. There, it is subjected to very intensive grinding by small ferromagnetic rods moved by rotating electromagnetic field. When the material particles are small enough, they are carried upwards in an air stream and pass two classifiers which separate too coarse material from the final product. The former constitutes a stream of recycle material and is re-ground; the latter is collected in a tank at the output of a cyclone. Air flow in the system is caused by underpressure generated with a blower located near the exhaust of the installation. The air flow in specific elements of the system, such as the mill chamber, recycle stream and classifiers, is controlled with butterfly dampers positioned by electric rotary actuators. The whole installation is equipped with numerous sensors and controlled with a PLC (Programmable Logic Controller) and SCADA (Supervisory Control And Data Acquisition) system.

The transport air plays a key role in the operation of this grinding circuit^22,23^. Most of all, appropriate air flow suspends the raw material in the working chamber of the mill. With an air flow too slow, the material would fall to the bottom of the working chamber and clog it. On the other hand, too fast flow of air would prematurely blow the material particles away from the mill chamber, which would result in exaggerate recycle, decreased material throughput and inefficient operation of the whole system. Moreover, the coarse material particles in the recycle stream need proper air flow to be moved along the pipeline and then raised towards the mill chamber. Additionally, the precise classifier (of inertial-impingement type in this installation^25^) requires higher air flow rates than the working chamber of the mill. Thus, extra air needs to be supplied just below the classifier (see Fig. 1a), and the air damper associated with it is never fully closed. Also, effectiveness of the separation process depends on the exact value of air flow rate through the classifier^25^.

The above three key air flow rates cannot be measured directly. This is due to moving material particles, which pose a serious risk to measurement equipment, and due to the shapes and dimensions of the installation elements (areas of stabilized air flow are not achievable there). Instead, the air flow is measured in the three inlet streams: main, recycle and additional (see Fig. 1a). Then, from their sums the key air flows are estimated^22^. These three inlet streams may be controlled by positioning of the associated butterfly dampers. This task is difficult, though, due to physical couplings between the air streams—they share a common intake, then they are separated to be joined again below the mill and below the precise classifier (see Fig. 1a). Also, operating characteristics of the dampers are nonlinear^22,23^.

Summarizing, the pneumatic system is multidimensional, unstable in the open loop, cross-coupled and nonlinear. This makes it a challenging plant for control, and requires a model to enable design and parametrization of a well-performing control algorithm. Also, there is a need for a simulation environment based on this model. This way, control scheme candidates may be evaluated first in simulation, and then only the best ones are be implemented in hardware for final tests on site, tremendously saving time, effort and costs.

### Identification experiment

The experiment aimed at identifying air flows behaviour in response to changing damper positions. Only clean air was used in the experiment, without the raw material or grinding elements. The latter clearly affect the air flow values by introducing extra pneumatic resistance, however, they involve too many influencing factors to be tested in a single experiment (composition, throughput, particle size, moisture content etc. of the material; amount, size, shape of the grinding media; rotational frequency of the electromagnetic field). Thus, it was advisable to create a “baseline” characteristics by using clean air only and testing numerous damper positions. The influence of other factors should be tested in separate experiments, probably under a limited set of damper positions—to save time and raw material. These results may be used to modify the “baseline” clean-air models according to current conditions, similarly as in^24^.

During the tests, the grinding installation (Fig. 1) was arranged as follows: the input material container was empty, but sealed air-tight, similarly as a pile of granular material would block the inflow of air. Screw feeder and mill inductor were switched off, and humidifier was disconnected, as not needed. The working chamber of the mill was empty (no raw material nor grinding media were present). However, both rotary valves were switched on, even though they did not convey any material. This was for the rotary valves to have similar air tightness as during the standard operation of the installation.

In the identification experiment, a series of step changes was performed on the position of one damper, gradually from closed to open and then gradually back to closed, at all possible combinations of the positions of the two remaining dampers. Three experiment runs were performed, each with a different damper being the most often repositioned one. Each step response was recorded for 40 seconds, during which the output signals settled, and then the next step change followed. Every damper could be positioned at 0–100% opening with 1% increments. The actually used damper positions were selected based on preliminary experiments that revealed the approximate shape of the steady-state characteristics. These finally tested damper positions were more densely spaced in regions of bigger curvature of the static characteristis, and more sparse in the flat areas of the characteristics (to save time during the experiments, as it was growing exponentially with each tested value). The following positions ${x}_\bullet$ were used:

- For the additional damper: $x_{\text {a}}$ = {10, 20, 30, 40, 50, 70, 99} [% open];
- For the main damper: $x_{\text {m}}$ = {0, 10, 15, 20, 30, 40, 50, 99} [% open];
- For the recycle damper: $x_{\text {r}}$ = {0, 10, 15, 20, 30, 50, 99} [% open].

The collected output signals included air speed at pipe axis *v*, relative pressure *p* and air temperature *T* at the end of each inlet pipe. The air speed *v* was later transformed to air mass flow *q* using the other collected quantities, as explained below. The measured and calculated signal values are listed in the supplementary data file.

Air velocity and air temperature were both measured with Delta OHM HD2937T01 transmitter^26^, one for each inlet pipe. Measurement range for air speed was set to 0.2–10 m/s, resulting in accuracy of ±(0.5 m/s + 3% of measurement). Integration time was selected as slow because of probable turbulences, as recommended by the manufacturer. Temperature measurements used a fixed (unselectable) range of −10 to +60 $^\circ \textrm{C}$ at ±0.3 $^\circ \textrm{C}$ accuracy. Relative air pressure was measured with ABB 264DS differential pressure transmitters^27^, whose H inputs were left unconnected (subject to atmospheric pressure) and L inputs were connected to the pipeline. The sensors were set to 0–8 kPa range (here meaning 0–8 kPa underpressure) and zero-calibrated at the start of the experiment.

Air mass flow *q* was calculated from the measurements in the following steps:

- Air density was^28^:
  1 $$\begin{aligned} \rho = \frac{ ( p + p_{\text {atm}} ) \cdot M}{R \cdot T} \,, \end{aligned}$$
  where atmospheric pressure was assumed with reasonable accuracy as $p_{\text {atm}} = 1013$ hPa; the universal gas constant was $R = 8.31446$ J/(mol K); and molar mass of dry air was used: $M = 28.97$ g/mol, as the difference caused by nonzero air humidity was not significant for the further calculations.
- Dynamic viscosity of air was approximated with^29^:
  2 $$\begin{aligned} \mu = 2.791 \cdot 10^{-7} \cdot T^{0.7355} \,. \end{aligned}$$
- Mean air speed in pipe cross-section was:
  3 $$\begin{aligned} w = c \cdot v \,, \end{aligned}$$
  where *c* was a dimensionless proportionality factor dependent on the flow regime, or Reynolds number $\textrm{Re}$ (explained later):
  - For laminar flow ($\textrm{Re}< 2000$), $c = c_{\text {laminar}} = 0.5$ (see p. 357 in^30^).
  - For turbulent flow ($\textrm{Re}> 4000$), *c* is higher and also, it grows with increasing Reynolds number. For simplification, this research used a constant approximation of $c = c_{\text {turbulent}} = 0.8$ for all turbulent flows. It was justified since Reynolds numbers finally estimated from the measurements did not exceed $\textrm{Re}= 28,000$, which meant the *c* values for these turbulent flow cases ranged from about 0.79 to about 0.82 (see p. 367 in^30^).
  - For transitional flow ($2000< \textrm{Re}< 4000$), the formula for *c* was based on^31^. Firstly, a weight  $\alpha \in \left[ 0, \, 1 \right]$ was defined that specified how much the flow was laminar (see eq. (9) in^31^):
    4 $$\begin{aligned} \alpha = \frac{1}{ 1 + \left( \frac{\textrm{Re}}{2720} \right) ^9 } \,, \end{aligned}$$
    and then the *c* values for laminar and turbulent flow were combined, similarly as in eq. (1) in^31^:
    5 $$\begin{aligned} c = (c_{\text {laminar}}) ^{\alpha } \cdot (c_{\text {turbulent}})^{1-\alpha } = 0.5 ^{\alpha } \cdot 0.8^{1-\alpha } \,. \end{aligned}$$
    This function’s value is close to 0.5 for laminar flows and close to 0.8 for turbulent flows, so a single formula (5) may actually be used for all Reynolds numbers (there is no need to use three separate cases for three flow regimes).
- Reynolds number (see eq. (1.24) in^30^):
  6 $$\begin{aligned} {\textrm{Re}}= \frac{ \rho \cdot w \cdot D }{ \mu } \,, \end{aligned}$$
  with *D* being the characteristic length (for flow in circular ducts: the pipe’s inner diameter)^30^. It was $D=102.3$ mm in this case.
  Reynolds number (6) depends on average velocity *w* (3), which uses the proportionality factor *c* (4–5), which again depends on Reynolds number. This loop of dependencies was solved iteratively for each data point, starting from the initial value of $c = (c_{\text {laminar}} + c_{\text {turbulent}})/2$, and then calculating $w,~\textrm{Re},~\alpha ,~c$ in a loop until the new estimation of $\textrm{Re}$ did not differ much from the old one, that is, $\frac{ \left| \textrm{Re}_{\text {new}} - \textrm{Re}_{\text {old}} \right| }{ \textrm{Re}_{\text {old}} } \leqslant 0.001 \,$. Such tolerance of 0.001 seemed reasonably small and also resulted in stable (converging) operation of the algorithm. Then, the final estimates of *c* and *w* were calculated from the newest value of $\textrm{Re}$.
- Volumetric flow rate of air was:
  7 $$\begin{aligned} Q = A \cdot w \,, \end{aligned}$$
  where $A = \frac{ \pi D^2 }{4} = 8219.4$ mm$^2$ was the pipe’s cross-sectional area.
- Mass flow rate of air was:
  8 $$\begin{aligned} q = Q \cdot \rho = A \cdot w \cdot \rho \,. \end{aligned}$$

### Data processing

**Figure 2:**
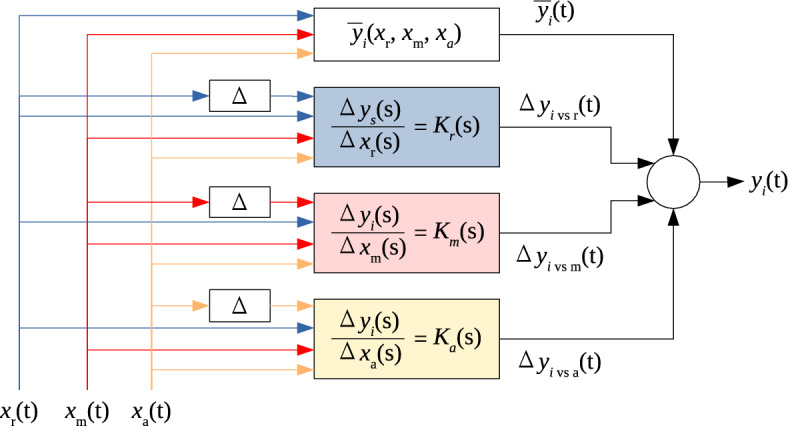
Structure of the model to be identified. Inputs $x_{\text {r}}$, $x_{\text {m}}$, $x_{\text {a}}$ are positions (openings) of recycle, main and additional air dampers; output $y_i$ is air mass flow or relative pressure at the end of given inlet stream $i \in \lbrace$r, m, a$\rbrace$; ${\overline{y}}_i$ is the steady-state characteristics of signal $y_i$; operator $\Delta$ indicates deviation from steady state; *s* is the Laplace variable; all parameters of transfer functions $K_i(\text {s})$ are dependent on the three inputs ($x_{\text {r}}$, $x_{\text {m}}$, $x_{\text {a}}$). Parameter estimation is performed separately for each output signal.

The model to be identified is schematically shown in Fig. 2. Its inputs are the positions of the three air dampers. The output is air mass flow or pressure at the end of one inlet pipe. If necessary, the mass flow rate could be transformed to volumetric flow rate or mean air speed, or centerline air speed, using the formulas introduced in the previous section. The figure shows model structure for only one output signal. Thus, in the complete simulation, such set of blocks is repeated six times—to calculate both air flow and pressure at each of the three pipes.

**Figure 3:**
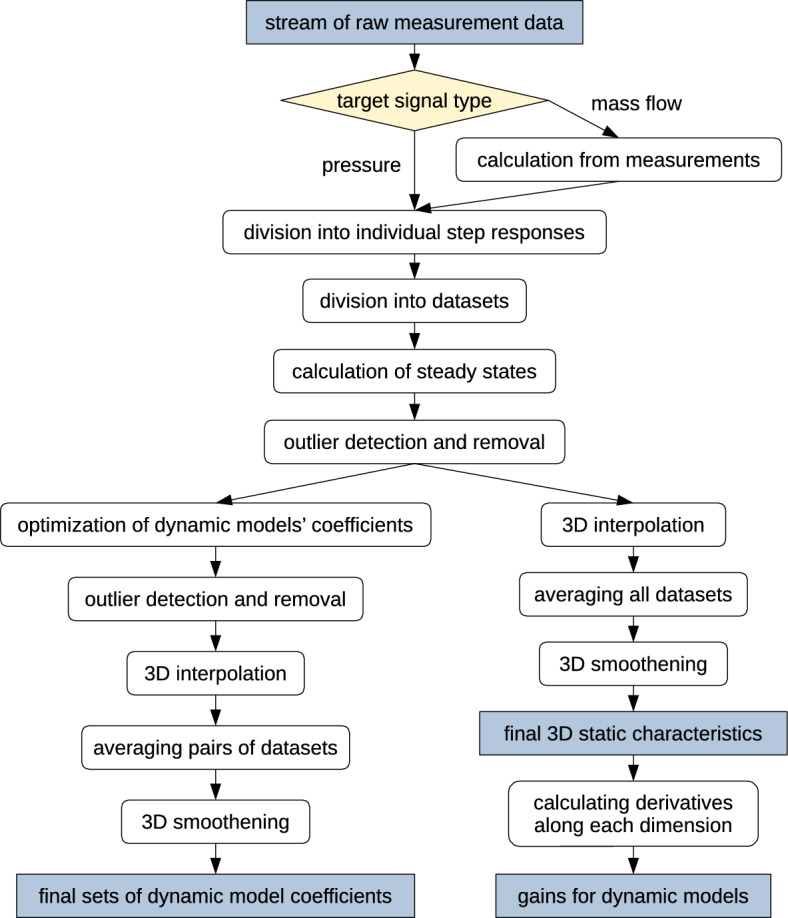
Stages of processing the measured data to estimate model parameters.

The model contains information on the steady state related to the current operating point {$x_{\text {r}}$, $x_{\text {m}}$, $x_{\text {a}}$} and three simple dynamic models which define output deviations from steady state in response to input deviations from steady state. The dynamic models’ parameter values depend on the operating point. All these dynamic and static coefficients need to be estimated from the measurements; the associated data processing stages are summarized in Fig. 3.

Air mass flows were estimated from the measurements, as explained in the previous section. Next, mass flow and pressure signals were split into individual step responses and then divided into six datasets per output signal. A separate dataset was associated with each air damper gradually opened or gradually closed.

Steady states of pressure and air mass flow after each input step change were determined, if possible (sometimes the measurements were too noisy or the air flow was so turbulent that the measured signals did not settle down during the observed time span). The steady states were determined from all step responses, so from all six datasets.

Incremental dynamic models $K_i(\text {s})$ were also identified. Only the damper positions that chaged the most often in the particular experiment run were used as dynamic model inputs; so, for a given pair of model input and model output signals, only two datasets were used, corresponding to the same input signal (damper position) increasing or decreasing. Initial values of model coefficients were estimated from the characteristic features of the analysed step responses, using similar methods as in^32–34^, but adapted to include time delay in the model, where applicable. Then, these rough estimates were refined by minimizing mean absolute error (MAE) between the actual and modelled signals. Based on the observed shapes of experimental signals, three model structures were tested: first order inertia with delay, second order system with and without delay (allowing both inertia and oscillatory systems). The first structure appeared to provide on average the best fit (in terms of MAE) or no worse than the others, while maintaining the most simplicity. Moreover, such structure of plant model is commonly used in controller tuning methods (see e.g.^35^), which would make the future controller parametrization easier. Thus, only first order models with delay were used in further stages.

Outlier detection methods were applied to all estimated coefficients. For steady states, the following algorithm was proposed:

1. Take the whole dataset (all steady states of one output signal, associated with one damper being only gradually opened or only gradually closed) as the initial dataset.
2. For each data point in the set:
  - Temporarily exclude this point from the dataset, forming a set of “known” points.
  - Calculate the “expected” value of the analysed data point: perform 3D linear interpolation on the known data. Interpolation procedure assumes that positions of the three air dampers are predictor variables, and the steady-state mass flow or pressure is the response variable. Linear interpolation was selected to ensure no artificial ripples in the interpolated hypersurface, and was found effective despite its simplicity.
  - Assess the quality of the analysed data point as an error between the actually measured and expected (interpolated) values. This error function was adopted as absolute difference, however, it could also be a more sophisticated one if needed. For example, error function could use weights related to reliability of interpolated output, and this reliability could be defined by number of points used to compute the interpolated value and their distances to the queried point.
3. Set a threshold on data point errors—97-th percentile of all error values, in this case (chosen experimentally)—above which a point is considered as probably outlying.
4. These outlying points could have biased the interpolation of expected values of their neighbours, so repeat the whole procedure, still with the complete dataset as the initial one (in point 1), but with only the probable inliers in the “known points” dataset (in point 2a).
5. Compare the indices of probably outlying data points that were found in this and in previous iteration of the algorithm. Continue iterating until the algorithm converges (the same indices are selected after each iteration) or until the selected indices cycle between two unchanging sets of values.
6. The outliers are finally assumed as the points indicated by the converged algorithm, or as union or intersection of the two alternating sets of points. This research adopted the more cautious case of union of the two sets.

In the case of dynamic models, the outlying ones were assumed as satisfying any of the following conditions:

- The associated steady-state value was marked as outlying,
- MAE of the dynamic model scaled by the value range of associated step response was above the adopted threshold (95-th percentile, in this case—chosen experimentally),
- Any model parameter—gain, time constant or time delay—was beyond the 95% of most commonly occuring values for this parameter (i.e., any parameter value was outside the possibly narrowest histogram fragment that contained at least 95% of all parameter values).

The parameter sets and static characteristics were meant for use in plant simulation and in design and tuning of control algorithms. Thus, it was necessary to interpolate (or extrapolate) their values into the whole range of damper positions that is used in normal operation of the grinding circuit. This was assumed as 0–100% opening for main and recycle dampers, and 10–100% opening for the additional damper, as the latter should never be completely closed to provide enough air flow through the classifier^22^. All these ranges contain positions in 1% increments, since such positions are settable on damper actuators. Several methods for multidimensional interpolation of scattered data were tested, to provide hypersurfaces of desired smoothness, appropriate for the considered kind of data. Finally, the datasets (with outliers removed) were extended with artificially added points in the flat areas of the static characteristics, to preserve this flatness during the final interpolation. Namely, in the regions where $x_{\text {a}} \in \left[ 70, \, 99 \right]$, or $x_{\text {m}} \in \left[ 50, \, 99 \right]$, or $x_{\text {r}} \in \left[ 50, \, 99 \right]$, points were added with 10% increments in damper position, and their output signal values were linearly interpolated (in three dimensions) from the existing data. Then, the main 3D interpolation of static characteristics was performed using thinplate radial basis functions (RBF)^36^. This method properly preserved the smooth curvature of the characteristics while keeping the introduced ripples (artifacts) to the minimum. For the temporal parameters of dynamic models, 3D linear interpolation was sufficient, as smoothness of these hypersurfaces was not that essential; and gains identified from step responses were not used further, for reasons that will be explained below.

Experiments with dampers being both opened and closed have shown a slight hysteresis in damper operation. This is probably due firstly to the operation of damper actuators, which maintain their own feedback loops when putting the dampers to the requested positions. The actual position usually slightly differs from the requested one, and this error changes upon each repositioning. Secondly, the rubber seal around the damper’s disk is somewhat flexible and differently affects the size of the pipe clearing when it is moved to position *n* from an opening higher or lower than *n*. In the future this hysteresis may be taken into account in the model; now, however, to simplify the overall model structure, this hysteresis was neglected and approximated with the mean of the individual characteristics. For steady-state values, all six interpolated characteristics were averaged. The dynamic model parameters were averaged from two datasets associated with the appropriate input signal (damper position) growing or decreasing.

Next, 3D smoothening filters were applied to the averaged interpolated data. A 3D box filter was found proper. This operation removed the unevenness which was due to measurement uncertainties propagated through the processing stages. It also eliminated any slight ripples artificially introduced by RBF interpolation into some nodes of the static characteristics. Great smoothness of the latter was especially important, as directional derivatives were calculated from them along all three dimensions, and any disturbances would be significantly amplified during the differentiation process. The directional derivatives provided the gains for dynamic models. This estimation method was preferred from getting the gains in a similar way as the other dynamic parameters, because this way the gains were exactly compatible with the static characteristics. Also, this meant the gain estimates resulted from combining all six datasets instead of just two, which made them more reliable.

### Model implementation in the simulator

The complete model of one output signal $y_i$ (air mass flow or pressure at the end of one inlet pipe) consists of four elements: static characteristics ${\overline{y}}_i = f \left( x_{\text {a}}, \, x_{\text {m}}, \, x_{\text {r}}\right)$ and three incremental dynamic models $\frac{\Delta y_{i}(s)}{\Delta x_{\text {X}}(s)} = \frac{k_i}{1+sT_i} e^{-s T_{0,i}}$, one per each damper’s position $x_{\text {X}}$. Symbol *s* stands for the Laplace variable. Symbols $k_i, \, T_i, \, T_{0,i}$ denote the gain, time constant and time delay of the identified dynamic model, and actually they are also functions of the operating point: $k_i = f \left( x_{\text {a}}, \, x_{\text {m}}, \, x_{\text {r}}\right)$, the same for $T_i$ and $T_{0,i}$. However, the implementation in code cannot be a simple sum of these four components; several adjustments are needed. They will be explained assuming step changes on damper positions, as these are easy to visualise and analyse. However, the simulator works for any type of excitation, as all signals can be composed of successive step changes—because in the real plant, the excitation (requested position of a damper) is issued by electronic hardware operating at specific sampling rate.

Firstly, it is necessary to either use the steady state value from the previous operating point $\left\{ x_{\text {a}}, \, x_{\text {m}}, \, x_{\text {r}}\right\}$ and then add the deviation(s) produced by the dynamic models; or, to immediately use the steady state value from the current operating point, but slow down its propagation to the output $y_i$ with the dynamics provided by the incremental models. The latter approach seemed easier to implement. So, on a step change of input $x_{\text {X}}$, the corresponding dynamic model should actually be excited with a square pulse of amplitude $-\Delta x_{\text {X}}$ and length equal to the model’s time delay at the current operating point. Taking into account that $k_i \cdot \Delta x_{\text {X}} = \Delta {\overline{y}}_i$, the dynamic model may be simplified to one having unit gain and excited directly with $-\Delta {\overline{y}}_i$, but excited only in these moments when damper X is moved, not the other dampers. Apart from code simplification, this substitution ensures that the initial output of the incremental model perfectly matches the change in the output of static characteristics block. So, the mentioned square pulse excitation cancels out the $\Delta {\overline{y}}_i$ change on output signal until time delay $T_{0,i}$ elapses, and then—thanks to time constant $T_i$ in the model—the old value of output signal slowly moves towards the new steady state.

In simulation it is necessary that the dynamic model output $\Delta y_i$ is smooth at the end (producing the inertial reaching of the new steady state), but sharp at the beginning (to ideally compensate the sharp change in steady state ${\overline{y}}_i$). It can be interpreted as the incremental dynamic model, which computes deviations from a steady state, is given a new value of steady state to base on. This is achieved by modifying the accumulator variable in the integrator part of the dynamic model: on start of each excitation by $-\Delta {\overline{y}}_i$, the accumulated value is also shifted by $-\Delta {\overline{y}}_i$. In consequence, the dynamic model produces a sharp edge at the output instead of its usual smooth transient.

Of course, the simulator correctly handles new excitations occurring before the system reaches steady state after the previous excitation. New square pulses are just added to the current input of dynamic model, and they are switched off after their individual durations elapse.

The last adjustment accounts for the situation when multiple (*N*) dampers are repositioned at the same time. Then, the new steady state is the effect of operation of *N* dynamic models. If each of them was excited with $-\Delta {\overline{y}}_i$, the total deviation produced would be *N* times bigger than necessary. Several solutions to this problem are possible, for example exciting each model with the corresponding $k_i \cdot \left( - \Delta x_{\text {X}} \right)$ instead of $-\Delta {\overline{y}}_i$, but this was not preferred, as was already explained. Moreover, there is a problem which value of $k_i = f \left( x_{\text {a}}, \, x_{\text {m}}, \, x_{\text {r}}\right)$ should be used in each of *N* dynamic models—that is, which ${x}_\bullet$ value should be used, old or new? Different selections would produce different transients, and it is hard to tell which version would be more proper. Alternatively, only one of *N* models could be excited, and it could be, for example, the slowest one; but this would again be only an approximation of the true situation. Finally, it was decided to excite each of *N* models in the already defined way, but only with 1/*N* of the usual excitation amplitude. This solution produces reasonable results and it was the simplest one to implement in code. Any discrepancies with the real plant behaviour should be negligible.

The sum of such defined steady state value ${\overline{y}}_i$ and three dynamic components $\Delta y_{i~vs~\text {a}}$, $\Delta y_{i~vs~\text {m}}$, $\Delta y_{i~vs~\text {r}}$ produces one output signal $y_i$ being the air mass flow or pressure in one inlet pipe. This structure is repeated three times to simulate all three mass flows, and if desired, next three times to also simulate the pressures. Of course, all component models use the same set of damper positions as their excitation, but they have separate static characteristics and dynamic parameters.

The whole model is simulated with a small time step of fixed size: 1/40 s (may be adjusted if needed). This is much faster than the usual values of time constants and time delays in the dynamic models (median values for all flow rate models are: $T_{\text {med}} = 1.44$ s, $T_{0,\text {med}} = 2.10$ s, and for pressure models: $T_{\text {med}} = 0.40$ s, $T_{0,\text {med}} = 0.81$ s). This is also 20 times faster than the control loops of the real grinding circuit, which are currently operating at 0.5 s period. Thus, the plant simulation is fast enough to emulate continuous time. Control part of the simulation environment was also prepared, but results of closed-loop tests will be analysed in a future publication. The simulated controllers are working with a discretized time step of 0.5 s (adjustable), and zero-order hold is included on the border from discrete to continuous time domain, both mimicking the operation of PLCs in the real installation.

The simulator was implemented in MATLAB Simulink software, and supported with a MATLAB script to load model parameters from disk, run the simulation and save the results to a file. Some open-loop simulations are presented in the next section.

## Results and discussion

Several scenarios were simulated to verify if the model was correctly implemented and its parameters properly estimated. Firstly, some verification tests showed if the simulated signals behaved as intended. Secondly, validation tests checked if the values of model outputs were similar to the data measured at the plant.

### Test 1: one damper at a time, waiting for steady states

**Figure 4:**
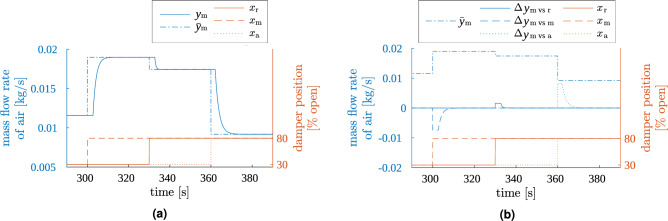
Result of test 1. Simulator output: air mass flow in the main stream (blue) for plant model excited with arbitrary step inputs (red). Only a fragment of the whole test is shown. (**a**) Total simulated air flow in the main stream $y_{\text {m}}$ compared to its steady-state value ${\overline{y}}_{\text {m}}$, (**b**) components of the simulated air flow, i.e., steady-state value ${\overline{y}}_{\text {m}}$ and deviations from it ($\Delta y_{{\text {m vs }} \bullet }$).

Firstly, a simulation was run in which the inputs (damper positions) were set to several arbitrarily chosen values. One damper was moving at a time. The output signals had enough time to settle down before new step change was issued on input. A fragment of the results is presented in Fig. 4.

The left panel of the figure shows the total output mass flow *y*. The output signals react to each step change on inputs, and with proper dynamics (1st order inertia with delay). Time delays and time constants of these responses vary with operating point of the system. The steady states actually achieved by signal *y* are equal to their theoretical values indicated by samples of static characteristics ${\overline{y}}$.

The right panel of the figure presents the components of each output signal, i.e. steady-state values and deviations from them produced by dynamic models. Each deviation signal $\Delta y_{\text {X}}$ indeed responds only to changes in its associated damper’s position $x_{\text {X}}$. After a step change in $x_{\text {X}}$, the appropriate dynamic model produces a response with maximum amplitude equal to the change in steady state ${\overline{y}}$, but with opposite sign. At the beginning, such responses sharply rise or fall. Then they stay constant for the duration of delay time (different for each operating point). Eventually, these responses settle down to zero, allowing the steady-state values to be fully reflected in the output *y*. All this behaviour is as was intended.

### Test 2: multiple dampers at once, waiting for steady states

Next verification step involved multiple dampers changing positions at once: first, they were changed pair-wise, and then all three simultaneously. For ease of result analysis, again the signals were let to settle down before a new set of step changes was issued.

**Figure 5:**
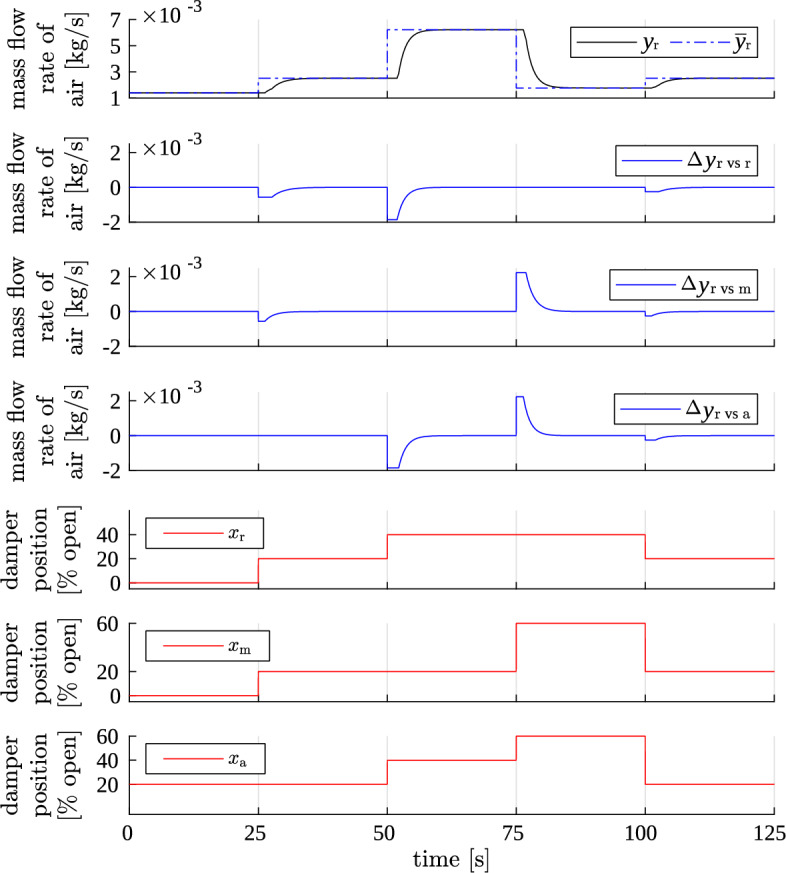
Result of test 2. Simulator output: mass flow of recycle air (blue) for plant model excited with simultaneous step changes on multiple inputs (red).

Simulation result is plotted in Fig. 5—only for the recycle air stream, as an example. The result was correct: each deviation signal $\Delta y_{\text {r vs } \bullet }$ responded to proper step changes; the total output signal $y_{\text {r}}$ had properly shaped transients and correct steady-state values.

### Test 3: not waiting for steady states

Another test verified if the simulator correctly handled new excitations that occurred during transient phase caused by a previous excitation. The test scenario involved:

- A step change on $x_{s1}$, then step change on $x_{s2}$ after 3 seconds, for several pairs of $\{s1,~s2\} \in \{ \text {a,~m,~r} \}$ (at simulation time 0–55 s);
- Successive step changes on all three dampers’ positions in 3 s intervals (at simulation time 55–80 s);
- Two successive step changes on position of the same damper, separated by 3 s interval (at simulation time 80–125 s);
- Multiple step changes on positions of all three dampers, occurring at the same moments on all three, with successive step changes separated by 1–3 s intervals (at simulation time 125–150 s).

**Figure 6:**
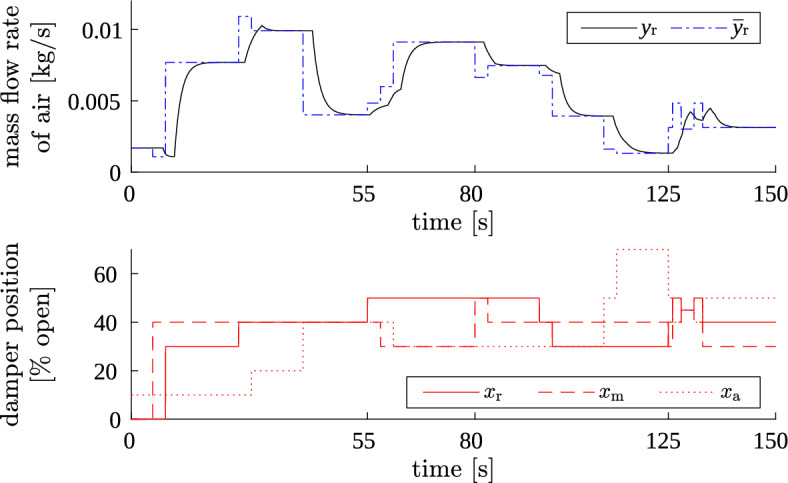
Result of test 3. Simulator output: mass flow of recycle air (top) for model excited with fast step changes (bottom).

After each of these stages, the signals were let to settle down to verify if correct steady-state value would be reached afterwards. Exemplary simulation result is shown in Fig. 6 (only for recycle air stream).

Transients were correctly shaped, and also appropriate steady-state values were achieved. So, the square pulses generation and the reset of integrators’ state variables (accumulators) in the simulator were well suited to operating in any conditions, not only in steady states. Consequently, this proved that the simulator may be used with arbitrary excitation signals, not only with infrequent step changes.

### Test 4: validation on data from identification experiment

**Figure 7:**
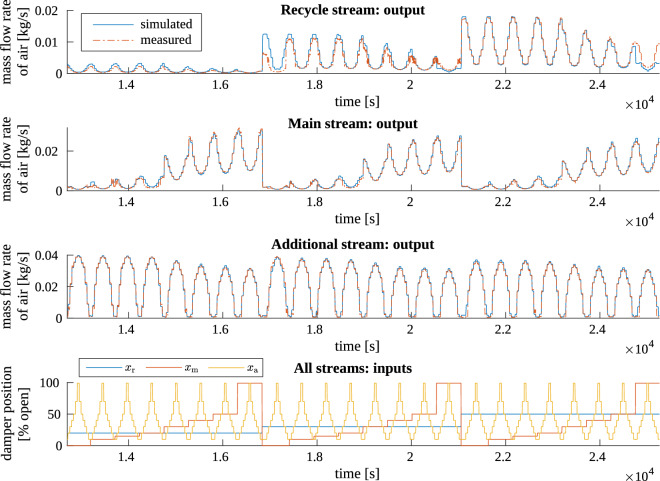
Result of test 4. Simulator output (mass flow rate of air in all inlet streams) compared to measurement results from experiment series no. 3: a broad fragment showing the steady states.

**Figure 8:**
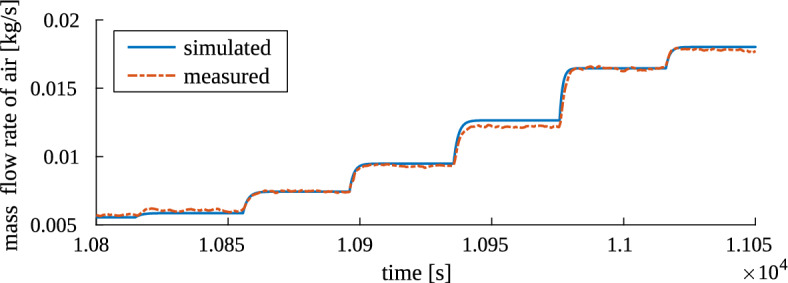
Result of test 4. Simulator output (mass flow rate of air in the main stream) compared to measurement results from experiment series no. 3: a small fragment showing the transients.

The simulator was excited with the same inputs as the real plant was during the identification experiment. Then, the measured and simulated signals were compared—in sense of both steady-state and transient values. Exemplary results are shown in Figs. 7 and 8, focusing on steady-state and transient results, respectively. These are the results for the third (last) experiment series, in which additional damper was changing position the most often, and recycle damper—the least often.

The plots (in Figs. 7, 8 and also from other experimental series, not presented here) show that the simulator works very good. The transients are quite well represented in simulation—regarding their shape, the delays and the rates of change. Also the steady-state values of the measurement data are generally very well reflected in the simulated output. Only for the recycle air flow rate, some big differences occurred for several operating points; otherwise the discrepancies were small. They were mostly caused by the fact that from one experiment series to the other, the steady states recorded for the same operating points of the installation were more or less varying. On the other hand, the static characteristics used in the simulator, calculated as in “Data processing” Section, averaged all of these measurements and differed (usually slightly) from the measured individuals.

## Summary

In this research, identification experiments were performed on the transport air subsystem of the grinding installation with electromagnetic mill. Maintaining the desired air flows in specific parts of the system is crucial for the efficiency of the grinding process, and even for its stability. For the three streams of inlet air in the installation, static characteristics and dynamic parameters were estimated for air mass flow and pressure reacting to changes in positions of controllable air dampers. Optimization and outlier detection mechanisms were involved. Then, the parameters were interpolated to the whole range of damper positions that might occur during normal operation of the grinding circuit. All estimated coefficients were combined into a single model and implemented in code. Implementation details are specified in this paper. Such constructed simulator was successfully verified on various artificial inputs and validated on the data from the identification experiment. In the next stage of research, the evaluated dynamic models and static characteristics are going to be used in design and tuning of air flow control schemes. Moreover, the simulator is going to provide a convenient testing environment for these control algorithms before they are finally verified on site.

## Supplementary Information

Supplementary Information.

## Data Availability

All data generated or analysed during this study are included in this published article (and its Supplementary Information files).
